# Size Of Gene Specific Inverted Repeat - Dependent Gene Deletion In *Saccharomyces cerevisiae*

**DOI:** 10.1371/journal.pone.0072137

**Published:** 2013-08-20

**Authors:** Chanyuen Lim, Annette Lin Luhe, Crystal Tear JingYing, Balaji Balagurunathan, Jinchuan Wu, Hua Zhao

**Affiliations:** 1 Industrial Biotechnology Division, Institute of Chemical and Engineering Sciences, Agency for Science, Technology and Research (A*STAR), Jurong Island, Singapore; 2 Process Sciences and Modeling Division, Institute of Chemical and Engineering Sciences, Agency for Science, Technology and Research (A*STAR), Jurong Island, Singapore; Duke University, United States of America

## Abstract

We describe here an approach for rapidly producing scar-free and precise gene deletions in *S. cerevisiae* with high efficiency. Preparation of the disruption gene cassette in this approach was simply performed by overlap extension-PCR of an invert repeat of a partial or complete sequence of the targeted gene with *URA3*. Integration of the prepared disruption gene cassette to the designated position of a target gene leads to the formation of a mutagenesis cassette within the yeast genome, which consists of a *URA3* gene flanked by the targeted gene and its inverted repeat between two short identical direct repeats. The inherent instability of the inverted sequences in close proximity facilitates the self-excision of the entire mutagenesis cassette deposited in the genome and promotes homologous recombination resulting in a seamless deletion via a single transformation. This rapid assembly circumvents the difficulty during preparation of disruption gene cassettes composed of two inverted repeats of the *URA3*, which requires the engineering of unique restriction sites for subsequent digestion and T4 DNA ligation *in vitro*. We further identified that the excision of the entire mutagenesis cassette flanked by two DRs in the transformed *S. cerevisiae* is dependent on the length of the inverted repeat of which a minimum of 800 bp is required for effective gene deletion. The deletion efficiency improves with the increase of the inverted repeat till 1.2 kb. Finally, the use of gene-specific inverted repeats of target genes enables simultaneous gene deletions. The procedure has the potential for application on other yeast strains to achieve precise and efficient removal of gene sequences.

## Introduction

Being the first eukaryotic organism to have its entire genome sequenced highlights the importance of *Saccharomyces cerevisiae* in biology [1], [2]. With its simple maintenance, quick growth, and genetically tractability, it is the ideal model eukaryotic microorganism contributing to our understanding of genetics, molecular and cell biology [3]. Already important in traditional biotechnology, this yeast has found wide applications in industrial biotechnology and synthetic biology or metabolic engineering strategies, through the altering and creating of new metabolic pathways [4], [5] to produce biofuels and value-added chemicals at reduced costs and waste generation. A more recent application is the hosting, sequencing and genetic engineering of prokaryotic genomes in their entirety as a yeast artificial chromosome (YAC) within the *Saccharomyces cerevisiae* cell [6] to circumvent the difficulty of culturing and the lack of simple genetic tools for these microorganisms.

Together with deletion studies which continue to yield new insights to the functioning of the cell, the fast pace of these new applications gives impetus to the rapid creation of *S. cerevisiae* strains with multiple precise and scar free deletions or alterations [2], [3], [7], [8]. The high recombinant frequencies in this organism are well suited and have indeed been expropriated for this purpose. Replacement of the target gene is achieved by insertion of the dominant resistance marker KanMX module containing flanking homologous sequences prepared using either PCR [9] or plasmid vectors [10], conferring resistance to the antibiotic G418 in the process. Subsequent deletions require the use of additional dominant selectable markers [11]–[12].

Removal or recycling of the already inserted marker was achieved using the loxP sequence specific Cre recombinase system [13]. This was further developed with the utilization of auxotrophic markers [14], the most prominent being the *URA3* uracil-FOA system [15]. In addition, the use of the low affinity lox71/66 sequences [16] reduced undesirable recombinations with the loxP sites of neighbouring deleted genes and potential genome perturbations in subsequent transformations. Despite the ease of creating these cassettes by PCR using similar primer sets, this Cre-mediated method requires a second transformation of a helper plasmid while leaving behind a loxP sequence or scar in the genome.

To overcome the above and the well-established but tedious meiotic recombination removal protocol [17], rapid scar-free marker recycling methods were developed using either a double transformations to pop/in and pop/out *URA3* modules [18] or by the inclusion of direct repeat sequences within the insertion cassette [19], [20] and the catalysis of their recombination using induced double stranded breaks [21]. Two recent iterations of this direct repeat mediated mutation are the MIRAGE method using a mutagenesis cassette containing a gnomically unstable directly inverted repeats of the *URA3* selectable marker gene [22] and a refinement of the double stranded break recombination based protocol [23], which contain the both the SECI homing endonuclease coding and its 18 bp recognition sequences.

We present here a simple and rapid approach for yeast gene deletion, where the disruption cassette consisting of the *URA3* marker gene and one copy of the inverted DNA sequence of a target gene is readily prepared by OE-PCR [24] prior to the replacement of the target chromosomal gene in a single transformation step, thus avoiding the time-consuming and inefficient restriction digestion-ligation steps required of MIRAGE cassettes [22] and the large mutagenesis cassette in TREC [23]. Deposition of the OE-PCR amplified disruption gene cassette into the designated position of a targeted gene locus leads to the formation of a mutagenesis cassette containing the *URA3* gene flanked by the previous partial/complete target gene and its inverted repeat within two short 25 bp endogenous direct repeats. The inherent instability of inverted repeats facilitates the excision of the entire mutagenesis cassette and promotes the homologous recombination between two direct repeats to obtain the scar-free deletion. We have also discovered that the length of the inverted repeat sequences played an important role in the success of the removal of the entire mutagenesis cassette. In addition, this method can be applied to simultaneously delete yeast genes. We applied this novel method to a number of genes in *S. cerevisiae*. It is demonstrated to be highly efficient.

## Materials and Methods

### Microorganisms Strains, Plasmids, Chemicals

*Saccharomyces cerevisiae* BY4742 (*MATα*; *his3Δ*1; *leu2Δ0*; *lys2Δ0*; *ura3Δ0*) was from EUROSCARF. The vector pYES2 were purchased from Invitrogen-Life Technologies and the high-fidelity Phusion polymerase from Thermo Fischer Scientific. The primers were synthesized by AITBiotech and listed in Tables S1 and S2 in Data S1 (Singapore) and 5′-fluoroorotic acid (5-FOA) from Zymo Research. All other chemicals were purchased from Sigma-Aldrich, unless otherwise mentioned. Yeast growth media used are YPD (1% yeast extract, 2% peptone, 1% Dextrose), synthetic complete media without uracil (SC-Ura), synthetic complete media without tryptophan and uracil (SC-Trp/Ura) and synthetic complete with 1 g/L 5-FOA (SC+FOA).

### Preparation of the Disruption Gene Cassette

The *URA3* marker gene was amplified from pYES2 using the respective N1 and N2 primer pairs as indicated in Table 1, while the gene-specific inverted repeat sequence was amplified from the *S. cerevisiae* genomic DNA using the respective N3 and N4 primer pairs. All the DNA sequences utilized in this study were the known sequences deposited previously in GenBank with the following accession numbers, *NDE1*, NP_013865; *NDE2*, NP_010198; *GUT2*, CAA86123; *GPD1*, CAA98582; *GPD2*, DAA10724; *GPP1*, P41277 and *GPP2*, P40106. To join the *URA3* marker gene and the gene-specific inverted repeat sequence together, the primers N1 and N5 were used in an OE-PCR for assembling the disruption gene cassette. These also avoid the use of extremely long oligos and allow the introduction of homologues arms to the OE-PCR product. The resulting PCR product was resolved by electrophoresis on 1% agarose gel and gel-purified with the Gel Purification Kit from Qiagen.

**Table 1:** Microbial strains, plasmids and oligos used in this study.

		Purpose	References
**Microbial strains**	*S. cerevisiae* BY4742	Host strain	EUROSCARF
**Plasmids**	pYES2	*URA3* template	Invitrogen
	pRS424	*TRP1* template	NEB
**oligos**	N1	With N2 for amplification of URA3 gene cassette	This study
	N2		This study
	N3	With N4 for amplification of gene-specific inverted repeat	This study
	N4		This study
	N5	With N1 for adding a homologous arm and OE-PCR assembly of disruption gene cassette	This study
	C1	Confirmation oligo forward	This study
	C2	Confirmation oligo reverse in the marker gene	This study
	C4	Confirmation oligo reverse	This study

### Yeast Transformation and Counter-selection by 5-FOA

The purified disruption gene cassette was then transformed into *S. cerevisiae* by a heat shock procedure [25] for homologous recombination *in* *vivo*. In brief, yeast was grown at 30°C in 45 ml YPD inoculated with 5 ml overnight starter culture with agitation at 200 rpm. The cells were harvested after 3–4 hours, elutriated twice by rinsing with sterile water and resuspended in 0.75 ∼ 1 ml of sterile water. 50 µl aliquots were then used for each transformation. The cells for each transformation were resuspended with 240 µl of 50% w/v PEG 3350, 36 µl of 1.0 M LiAc, 50 µl of 2 mg/ml salmon-sperm DNA and 34 µl of the prepared disruption gene cassette, followed by incubation at 42°C for 1 hour. After the transformation, the cells were pelleted and resuspended in water and plated on SC-Ura plates.

Single colonies were randomly picked for verification by colony PCR using the respective C1 and the C2 confirmation oligos on their emergence after 2–3 days of incubation at 30°C. These colonies were vortexed in 10 µl of 0.2% SDS, lysed at 98°C for 10 min and followed by micro-centrifugation. The resulting supernatant was used as the template for PCR amplification. Further verification was performed on the genomic DNA isolates. Once the correct recombination was verified, the colonies were inoculated into YPD+FOA medium and the resulting overnight culture were then spread on SC+FOA plate to counter-select for URA-negative colonies. Confirmation of the successful gene deletion was performed initially by yeast colony PCR using the respective C1 and C4 confirmation oligos. Genomic DNA was further isolated from the initially verified colonies for a second confirmation of the targeted gene deletion by PCR and subsequent DNA sequencing of the resultant PCR products.

## Results

**Figure 1:**
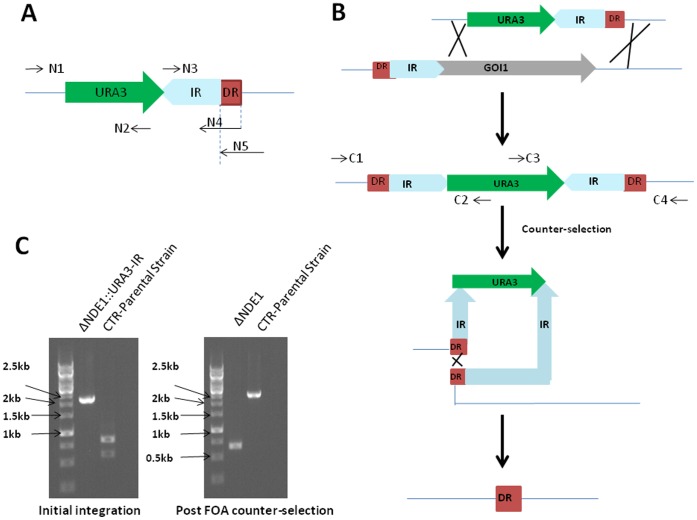
Scheme of the approach for gene deletion in *S. cerevisiae* using mutagenesis cassettes containing gene-specific inverted repeats. **A:** Preparation of disruption gene cassette by OE-PCR. N1 through N5 are oligos for amplification of individual DNA fragments and the final disruption gene cassette. **B:** The procedure for rapid and scar-free gene deletion. The disruption cassette consisting of the *URA3* marker and one copy of the inverted DNA sequence of a target gene is created by OE-PCR and flanked by 36 bp homologous arms. C: Deletion of *NDE1* gene by the described approach in this study. CTR: parental *S. cerevisiae* strain; IR: inverted repeat; DR: direct repeat; GOI: gene of interest.

The approach developed here is based upon the inherent instability of inverted repeat sequences in the genome of microorganisms. As depicted in Fig. 1A, the inverted sequence was selected as either partial or a complete DNA sequence from a target gene, while the optimum direct repeat (DR) size was adopted from a previous study [22] and designated to be 25 bp immediately upstream of the target gene. The disruption cassette consisting of the *URA3* marker and one copy of the inverted DNA sequence of a target gene is created by OE-PCR and flanked by 36 bp homologous arms. Deposition of the OE-PCR amplified disruption gene cassette into the designated position of a targeted gene locus leads to the formation of a semi-heterologous mutagenesis artifact in the genome of *S. cerevisiae*, which consists of the *URA3* gene flanked by the previous target gene and its inverted repeat (Fig. 1B). The genomic instability of this resultant mutagenesis artifact facilitates the precise self-excision of the entire mutagenesis cassette and promotes the homologous recombination between the two DRs. The *URA3* marker gene is efficiently removed during counter-selection using 5-FOA to obtain the scar-free deletion.

To verify the correct integration of the disruption gene cassette, the primer C1 corresponding to a few hundred bp upstream of the target sequence and C2 priming the middle part of *URA3* marker were used (Fig. 1B). If further confirmation was necessary, C3 priming of the URA3 gene can be used in conjunction with C4, a few hundred bp downstream of the target gene. The colonies with correct integrations were counter-selected for uracil auxotrophy, in a 5′-FOA-promoted dislodgment of the disruption cassette to form a scar-less deletion. Final verification of the deletion event is performed by using the primer pairs C1–C4 (Fig. 1B), which would result in a truncated and exact PCR product for the mutant in comparison to its parental strain.

To prove the concept, we first applied this method to delete *NDE1* in *S. cerevisiae.* Using OE-PCR, the disruption gene cassette was prepared by fusing the *URA3* selectable marker gene with a 1 kb partial inverted sequence of *NDE1* and transformed the resulting PCR product into yeast. PCR examination of the resulting transformants showed that the frequency of the correct integration of the disruption gene cassette to the target gene locus was ca. 50%. As indicated in Fig. 1C, we obtained the expected 2234 bp PCR product with the confirmation oligos C1 and C2. The subsequent counter-selection with 5-FOA led to the excision of the entire mutagenesis gene cassette including the *URA3* marker and its flanking inverted repeats and left with one copy of the DR. The successful scar-free deletion of *NDE1* gene yielded the predicted 602 bp sized PCR products with the confirmation oligos C1 and C4 (Fig. 1C) and by DNA sequencing of the target mutated allele. Although >1 Kb PR present in the disruption cassettes is identical to the target gene and has higher integration rate to the target gene *per se*, this integration would not deposit an inverted repeat of the target gene onto the chromosome and could not produce the entire mutagenesis gene cassette (defined as the selectable marker gene flanked by two inverted repeats). Therefore, it is not a successful integration and will be ruled out as wrong integration by initial screening using diagnostic colony PCR with the confirmation oligos C1 & C2.

In consideration of easier preparation of the disruption gene cassette by PCR-overlapping a shorter gene-specific inverted sequence with the *URA3*, We next asked if shorter inverted repeat lengths are effective in gene deletion. We attempted to fuse a 400 bp inverted sequence of the partial *NDE1* gene with *URA3* and transformed the resulting disruption gene cassette into *S. cerevisiae*. The disruption gene cassette successfully targeted at the *NDE1* locus accordingly. However, counter-selection of the resulting transformants with 5-FOA was not successful and no 5-FOA resistant colony was observed on SC+FOA plates. Although in a few cases, we managed to obtain a few colonies resistant to 5-FOA, diagnostic PCRs of the resulting colonies indicated the existence of mutated, unfunctional *URA3* allele in the disruption gene cassette deposited in the designated position of yeast genome by 5-FOA counter-selection (data not shown). In comparison with the previously efficient gene deletions with 1 kb inverted sequence of the partial *NDE1* gene, the results presented here acutely suggests that the size of the inverted repeat sequence affects the efficiency for the subsequent excision of mutagenesis cassette. This prompted us to systematically investigate the size effect of the inverted repeat sequence. By employing disruption cassettes containing various lengths of the inverted repeat of the partial/complete *NDE1* gene cassette ranging from 400 bp to 1.6 kb, we examined their gene deletion efficiencies. It is observed that the successful integration of the individual disruption gene cassettes to the targeted *NDE1* locus were ca. 50–90%. However, the subsequent removal of the entire mutagenesis gene cassettes flanked by DRs is dependent upon the size of the inverted repeat. As indicated in Table 2, with the inverted repeat sequence below 800 bp, no 5-FOA resistant colonies were obtained. Successful excision of the entire mutagenesis gene cassettes in 5-FOA counter-selection was observed using the 800 bp length inverted repeat and the efficiency increased with the increase of the size of inverted repeat up to 1.2 kb. No further increased efficiency was detected beyond 1.2 kb.

**Table 2:** The impact of various lengths of inverted sequence on *NDE1* gene deletion efficiency.

Size of inverted repeat (kb)	Correct initial integration rate*	Successful excision rate*
0.4	70%	0
0.6	90%	0
0.8	70%	70%
1	50%	90%
1.2	60%	100%
1.4	60%	100%
1.6	50%	100%

* The success rate was based on the PCR analysis of 10 colonies randomly selected from the selective plates. The correct initial integration of the disruption gene cassette was analyzed by PCR amplification with the confirmation oligos C1 and C2, and the excision rate was based on PCRs using the confirmation oligos C1 and C4.

We next examined the robustness of this gene deletion approach in *S. cerevisiae*. We randomly selected a few genes to test their deletion efficiencies. Using 1.2 kb lengths of gene-specific inverted repeat of each individual target gene, the disruption gene cassettes were prepared by overlapping each with the *URA3* encoding sequence and transformed separately into *S. cerevisiae*. Post 5-FOA selection, the resulting colonies on SC+FOA plates were subjected to PCR analysis with the corresponding confirmation oligos C1 and C4 to verify the successfully looping out of the entire mutagenesis cassettes flanked by the individual DRs. As summarized in Table 3, the individual gene targets were successfully replaced by the disruption gene cassette with 50–80% correct integration rate and 100% successful excision rate post 5-FOA counter-selection. The resulting PCR products amplified with the confirmation oligos C1 and C4 were further subjected to DNA sequencing. Consistent with earlier observations, the entire mutagenesis cassettes including the *URA3* maker gene flanked by two gene-specific inverted repeats were excised from yeast genome, leaving one copy of the DR. The results here confirm the scar-free and precise gene deletion events and positively demonstrate the effectiveness of the current approach on gene deletion in yeast.

**Table 3:** The success rate of gene deletion using ca. 1.2 kb inverted repeat of individual gene.

Targeted parental allele	Correct initial integration rate*	Successful excision rate*
*NDE1*	60%	100%
*NDE2*	60%	100%
*GUT2*	70%	100%
*GPD1*	80%	100%
*GPD2*	60%	100%
*GPP1*	70%	100%
*GPP2*	50%	100%

* The success rate was based on the PCR analysis of 10 colonies randomly selected from the selective plates. The correct integration was analyzed by PCR amplification with the confirmation oligos C1 and C2, and the excision rate was based on PCRs using the confirmation oligos C1 and C4.

* In cases where the size of a targeted gene is below 1 kb, its flanking sequences together with the structure gene were amplified as a 1.2 kb inverted repeat.

**Figure 2:**
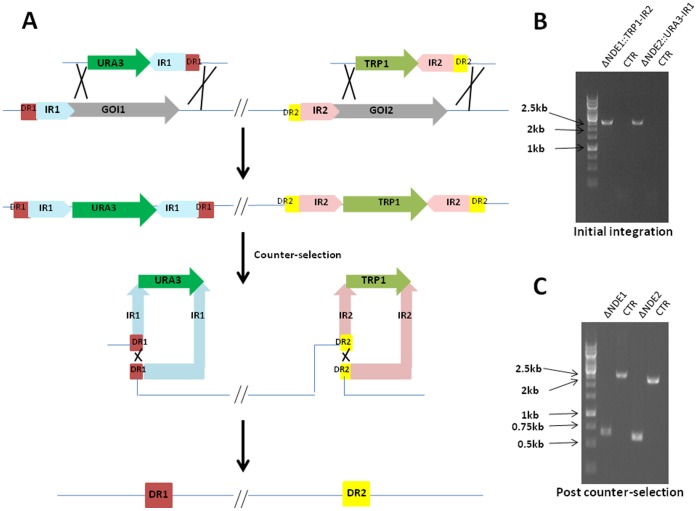
An illustration of simultaneous gene deletions in *S. cerevisiae*. **A:** Preparation of disruption gene cassettes by utilizing prototrophy for the nucleotide uracil and tryptophan and counter-selection for their respective auxotrophs using 5′-FOA and 5-FFA. **B:** Initial confirmation of the integration of the disruption gene cassettes *TRP1-irNDE1* and *URA3-irNDE2* into *NDE1* and *NDE2* loci, respectively for simultaneous gene deletion with C1 and C2 confirmation oligos and genomic DNA isolates. **C:** PCR-confirmation of excision of the disruption gene cassettes post counter-selection using C1 and C4 oligo pair. CTR: parental *S. cerevisiae* strain; IR: inverted repeat; DR: directed repeat; GOI: gene of interest.

In addition to its effectiveness of the current approach on individual gene deletions, we proceeded to examine the simultaneous deletion of *NDE1* and *NDE2* in *S. cerevisiae*. Two disruption gene cassettes were prepared using OE-PCR of 1.2 kb of the inverted repeats of partial *NDE1* and *NDE2* with the *TRP1* and *URA3* selectable marker gene cassettes, respectively. The resulting disruption gene cassettes, *TRP1-irNDE1* and *URA3-irNDE2*, were co-transformed in *S. cerevisiae*. Post transformation, yeast cells were spread onto selective plates (SC-Ura/Trp) and PCRs were performed with genomic DNA isolates from those transformants for analyzing the correct integration of the disruption gene cassettes into *NDE1* and *NDE2* gene loci. We observed a 20% success rate for integration of both disruption gene cassettes into their designated positions in yeast genome. After the initial confirmation, the mutants with both the *NDE1* and *NDE2* loci replaced by those disruption gene cassettes were counter-selected in both 5-FOA and 5-Fluoroanthranilic Acid (5-FAA). As indicated in Fig. 2B & C, the selected resistant mutants yielded the expected 602 and 550 bp PCR products respectively by using the confirmation oligos C1 and C4. By contrast, the parental *S. cerevisiae* strain with the intact *NDE1* and *NDE2* indicated 2300 and 2200 bp PCR products, respectively. These results demonstrated that the *NDE1* and *NDE2* genes were simultaneously deleted from the parental *S. cerevisiae* strain albeit at lower success rate for initial integration of the two disruption gene cassettes in the targeted loci.

## Discussion

Due to their propensity to form motifs [26] through self base pairing, inverted repeats are unstable in the genome. This intra-strand annealing leads to the disruption of the functionally essential double stranded B-DNA structure. Evidence suggest the formation and resolution of these motif structures occur during DNA replication [27], beginning with the slippage or dislocation of the primary DNA polymerase followed by breakage, re-annealing of the DNA and reattachment and continual activity of the polymerase. This chromosomal fragility is utilized in this application, to catalyze breakage and in doing so bring two direct repeats in close proximity for homologous recombination to occur.

Homologous recombinations between direct repeats have been previously used to achieve scar-free marker recycling [19], [22], [23], [28]. Although homologous recombination resolution of arrested replication has been implicated in gene disruption, intra-chromosomal transfers [29] and other genomic perturbations [30], two previous studies [22], [31] reported that motif resolution and removal by homologous recombination takes precedence over other breakage repair mechanisms in close proximity of short direct repeats, using the expected sizes of diagnostic PCR products. In our study, the inverted sequence was excised under 5-FOA selection with high frequency and imprecise DNA repair due to end joining between two direct repeats or within the inverted sequences is seldom observed.

In this study, we determined that the size of the inverted repeats played an important role in the excision of the entire mutagenesis cassettes. While short adjacent inverted repeats can be PCR amplified, longer adjacent inverted sequences however cannot be amplified through PCR due to the high susceptibility for template self annealing and the formation of aberrant PCR products [27], which was verified in the previous study for the disruption cassette preparation [22]. With this in mind, we identified that a minimal 800 bp of a inverted repeat is essential for gene deletion in this approach and is sufficient with a 1–1.2 kb inverted repeat. This also supports the observation that the formation and maintenance of stable cruciform structures are prerequisites for its removal [32]. The inclusion of the inverted repeat sequence within the mutagenesis cassette also serves as an additional buffer against undesired genomic insertions. The inability to form hairpin loops through self-base pairing results in retention of the *URA3* gene and non-viability of these yeast cells under 5-FOA counter-selection.

Inverted repeats have been previously employed for yeast gene deletions. The major limitation however, is its difficulty in the preparation of the disruption gene cassette as demonstrated in MIRAGE [22], which consists of two directly inverted repeats of *URA3* marker gene. Due to the inability to PCR amplify the inverted repeat sequences, appending of the two inverted repeat sequences prepared separately by PCR for the preparation of disruption gene cassette must involve restriction digestion and T4 DNA ligation. The inefficiencies in T4 DNA ligation frequently leads to the failure of the preparation of sufficient invert repeated disruption gene cassette. In addition, most ligation reactions are dominated by the self-ligation products. This creates tandem repeats of the *URA3* gene which cannot be used for gene disruption. This method is inefficient. In our experience, it took us around a month to successfully remove a gene by using this previously developed method.

By contrast, a major feature of our approach is its simpler and rapid preparation of disruption gene cassettes at high quantities. The use of the gene-specific inverted repeat enables the easy preparation of disruption of gene cassette by PCR-overlapping a copy of the inverted sequence of a target gene with a selectable marker gene. The resultant mutagenesis cassette is excised at high frequencies as with MIRAGE, achieving marker recycling easily in a single transformation. This is an improvement over previous methods using unaided homologous recombination of DRs [19] and do not require a second transformation [18], [33] of a pop out cassette, which was reported to be at lower frequencies.

Unlike other recent methods requiring the use of endonuclease mediated double strand breakage [23] no additional functional expression of exogenous endonucleases or cellular mechanisms in the form of helper plasmids or insertion cassettes are required beyond that of *URA3*. This allows deletions to be carried out in growth impaired strains without the need for growth and subsequent selection in permissive media. A scar-free or clean deletion was achieved in this example as compared to the Cre/loxP recombinase driven marker cycling methods [13], [14]. By applying this method, we were able to successfully delete a gene in one week. Furthermore, the employment of the inverted repeats capable of promoting the homologous recombination frequency in yeast finally leads to gene deletion with high efficiency in comparison with other PCR-based methods for gene deletion in *S. cerevisiae* [9], [23]. The suggested occurrence of imprecise deletions involving the use of invert repeats due to replication slippage [23] was not significant in this study. Furthermore, precise deletions of the target sequences are confirmed easily by the size of the expected PCR products.

The capacity for simultaneous multiplex gene deletion is also demonstrated in this study. By using the appropriate counter-selectable markers, simple preparation of two or more disruption gene cassettes by OE-PCR enables the simultaneous gene deletion. As with other reported procedures for gene deletions [33], [34], this protocol can be readily adjusted to produce sequence insertions and replacements in the genome. In addition, the higher efficiencies of precise marker excision with the gene-specific inverted sequences demonstrated here can also be easily adapted to other microorganisms for efficient gene deletion.

When working on the manuscript, the new approach based on the bacterial immune CRISPR/Cas9 system was published [35]. This method offers efficient genome editing in *S. cerevisiae* and requires the functional expression of Cas9 and the preparation of sgRNA in *S. cerevisiae*. The approach we present here offers an alternative for precise yeast gene deletion via the rapid preparation of disruption cassettes and involving a single transformation.

## Supporting Information

Data S1 (DOC)
