# Supplementary material for: Size Of Gene Specific Inverted Repeat - Dependent Gene Deletion In Saccharomyces cerevisiae
Source: PLoS One. 2013 Aug 20;8(8):e72137. doi: 10.1371/journal.pone.0072137 (PMC3748122; doi:10.1371/journal.pone.0072137)
Supplement: Data S1 — (DOC) [file pone.0072137.s001.doc]

**suppLEmentary data**

**Supplementary Table S1.** oligos used for disruption of *NDE1* gene in *S. cerevisiae* with various gene-specific inverted repeats.

| Name | Sequences (5’-3’) |
| --- | --- |
| N1_400_NDE1_for | AAA TCT TCC GCT AAG AAT AAC GAC TAC GAC TTG GAC TTG ATT TTT TAT TCT TTT TTT TGA |
| N2_400_NDE1 _rev | CGC TTT TGA AAA ATT TGG ACA CCA CGC AAA AAA GGG AAT AAG GGC GAC |
| N3_400_NDE1_for | GTC GCC CTT ATT CCC TTT TTT GCG TGG TGT CCA AAT TTT TCA AAA GCG |
| N1_600_NDE1_for | AGA TCC TGA GAG AGC AAG ATT GTT GAG CTT TGT TGT CGT TTT TTT TAT TCT TTT TTT TGA TTT C |
| N2_600_NDE1 _rev | CCT GAA AAC AAA ACA ATT AAG GTC CGC AAA AAA GGG AAT AAG GGC GAC |
| N3_600_NDE1_for | GTC GCC CTT ATT CCC TTT TTT GCG GAC CTT AAT TGT TTT GTT TTC AGG |
| N1_800_NDE1_for | ATG CTC AAG ATT TAT TCA AAG AGG AAA AAA TCG ATT TAA GTT TTT TAT TCT TTT TTT TGA |
| N2_800_NDE1 _rev | GAA AGC TGC CTC CCT ATC TCC AAA CGC AAA AAA GGG AAT AAG GGC GAC |
| N3_800_NDE1_for | GTC GCC CTT ATT CCC TTT TTT GCG TTT GGA GAT AGG GAG GCA GCT TTC |
| N1_1000_NDE1_for | TCA AGA CGT GGT TTG TTG ATA GAT AAC AAA CTT CAA CTT TTT TTT TAT TCT TTT TTT TGA |
| N2_1000_NDE1 _rev | TGT TTG ACA AGT ATC TCG TTG ACT CGC AAA AAA GGG AAT AAG GGC GAC |
| N3_1000_NDE1_for | GTC GCC CTT ATT CCC TTT TTT GCG AGT CAA CGA GAT ACT TGT CAA ACA |
| N1_1200_NDE1_for | AGA CGA TTC AGA AGT CGC TAG ATT AAA GAA CCA AAT AGT CTT TTT TAT TCT TTT TTT TGA |
| N2_1200_NDE1 _rev | *TTA GAG GAA CAG GAC* CGC AAA AAA GGG AAT AAG GGC GAC |
| N3_1200_NDE1_for | GTC GCC CTT ATT CCC TTT TTT GCG GTC CTG TTC CTC TAA TTT AGT CAT |
| N1_1400_NDE1_for | TGT GTC TAT CCT TTA GAA ACA GAG TTC TTG TCG CTA TGG ATT TTT TAT TCT TTT TTT TGA |
| N2_1400_NDE1 _rev | GAA CTG GAA AAT GAC CCA TGC TAA CGC AAA AAA GGG AAT AAG GGC GAC |
| N3_1400_NDE1_for | GTC GCC CTT ATT CCC TTT TTT GCG TTA GCA TGG GTC ATT TTC CAG TTC |
| N1_1600_NDE1_for | AGC AGA GGC CTT GTC CCT TTT TTA TGC TAA TAA AAT TAA ATT TTT TAT TCT TTT TTT TGA |
| N2_1600_NDE1 _rev | TCT GGA AAT CTG CTT ATT TGG CAA CGC AAA AAA GGG AAT AAG GGC GAC |
| N3_1600_NDE1_for | GTC GCC CTT ATT CCC TTT TTT GCG TTG CCA AAT AAG CAG ATT TCC AGA |
| N4_NDE1_rev | t att att ggt taa ttt ttt att tgc ATG ATT AGA CAA TCA TTA AT  (used for all deletions of NDE1 with various lengths of palindromic repeat) |
| N5_NDE1_rev | atc atc att taa aaa tgt tat tct ctt gta tct att tct ata tta ttg gtt aat ttt tta ttt gc  (used for all deletions of NDE1 with various lengths of palindromic repeat) |
| C1_Conf_delNDE1_for | gta tat ata aaa gag gtt gtg tta (for all deletion of NDE1) |
| C2_Conf_URA3_rev | CAT TCG TAA TGT CTG CCC ATT CTG (for all deletions) |
| C4_Conf_delNDE1_rev1 | ATT ATA CAT ACT TTA TTT CTC TTG G (for confirming the deletion of NDE1 using 0.4, 0.6, 0.8, 1 and 1.2 kb palindromic repeats) |
| C4_Conf_delNDE1_rev2 | GCA GGG AGT ATA AGC AAC GAT TG (for confirming the deletion of NDE1 using 1.4 and 1.6 kb palindromic repeats) |

**Supplementary Table S2. Oligos used for the disruption of various genes in *S. cerevisiae***

| Name | Sequence (5’-3’) |
| --- | --- |
| C2_Conf_URA3_rev | CAT TCG TAA TGT CTG CCC ATT CTG (same for all deletions) |
| N1_NDE1_for | AGA CGA TTC AGA AGT CGC TAG ATT AAA GAA CCA AAT AGT CTT TTT TAT TCT TTT TTT TGA |
| N2_NDE1 _rev | *TTA GAG GAA CAG GAC* CGC AAA AAA GGG AAT AAG GGC GAC |
| N3_NDE1_for | GTC GCC CTT ATT CCC TTT TTT GCG GTC CTG TTC CTC TAA TTT AGT CAT |
| N4_NDE1_rev | t att att ggt taa ttt ttt att tgc ATG ATT AGA CAA TCA TTA AT |
| N5_NDE1_rev | atc atc att taa aaa tgt tat tct ctt gta tct att tct ata tta ttg gtt aat ttt tta ttt gc |
| C1_Conf_delNDE1_for | gta tat ata aaa gag gtt gtg tta |
| C4_Conf_delNDE1_rev | ATT ATA CAT ACT TTA TTT CTC TTG G |
| N1_NDE2_for | GAA ATC TAA GTT GGA TAA GTT CAA CTA CAA GCA TAT GGG TTT TTT TAT TCT TTT TTT TGA |
| N2_NDE2 _rev | AAT GAC AAG TTG GAG CTT CTC GGT CGC AAA AAA GGG AAT AAG GGC GAC |
| N3_NDE2_for | GTC GCC CTT ATT CCC TTT TTT GCG ACC GAG AAG CTC CAA CTT GTC ATT |
| N4_NDE2_rev | C TAT ACG AAT GGC AAG TAC GGT GGT ATG CTG CCC AGA CTT GGT TT |
| N5_NDE2_rev | AAA CAA TTT GAA TTG GAA CAA TGA ATA TAT AAA ACA AGG ACT ATA CGA ATG GCA A |
| C1_Conf_delNDE2_for | TGC GGT ATT GCG TAC ACC TGT CAT |
| C4_Conf_delNDE2_rev | CGT AAA ATT CCA ACA GGC AAA CAG |
| N1_GUT2_for | AAT GGC TGA GGA AAC AGT CGA CAA AGT TGT CGA AGT TGG CTT TTT TAT TCT TTT TTT TGA |
| N2_GUT2_rev | GAT ATC TTG AAA GAA CTA CAG CAC CGC AAA AAA GGG AAT AAG GGC GAC |
| N3_GUT2_for | GTC GCC CTT ATT CCC TTT TTT GCG GTG CTG TAG TTC TTT CAA GAT ATC |
| N4_GUT2_rev | A TAT TAG TAC GTA TTT AGT CTT GTA ATG TTT TCG GTA ACG AGA AGA AGA GC |
| N5_GUT2_rev | GTG AAT GTT ATC TTT GTC ACC CTT AAC TAT CAT GAT CGA TAT ATT AGT ACG TAT T |
| C1_Conf_delGUT2_for | GCC AAG CGC AAG GGC ATC AGC GAG |
| C4_Conf_delGUT2_rev | GAA GTC TAC ACA ATG ATT ATA TAG |
| N1_GPP1_for | GGG GTC TTA ACA ACC TTG ATT TTG TTA GAA CGG GTG TTG TTT TTT TAT TCT TTT TTT TGA |
| N2_GPP1 _rev | CAATCT TTT GTT CTT CAA TCA AGA CGC AAA AAA GGG AAT AAG GGC GAC |
| N3_GPP1_for | GTC GCC CTT ATT CCC TTT TTT GCG TCT TGA TTG AAG AAC AAA AGA TTG |
| N4_GPP1_rev | T GCG ATG GTT TGT ATA TTT GCT TTT ATG CCT TTG ACC ACA AAA CC |
| N5_GPP1_rev | ctc cgc tgt tga aag att gaa atg aga taa tat tca ttg cga tgg ttt gta t |
| C1_Conf_delGPP1_for | ctt tgt gtc ttt taa ttt tga cca |
| C4_Conf_delGPP1_rev | gag ttt tca tga aaa ctt att atg |
| N1_GPP2_for | caa tac atc tct gtg caa tct gga tca ctt tgg ttg cct gTT TTT TAT TCT TTT TTT TGA |
| N2_GPP2 _rev | ccc aac tat aat gag gac tac aaa CGC AAA AAA GGG AAT AAG GGC GAC |
| N3_GPP2_for | GTC GCC CTT ATT CCC TTT TTT GCG ttt gta gtc ctc att ata gtt ggg |
| N4_GPP2_rev | t ccg aat att gtt ttt att gtt tta ATG GGA TTG ACT ACT AAA CC |
| N5_GPP2_rev | ggg ata atc ctc taa ggt taa act tga tgg aaa cat ttc cga ata ttg ttt t |
| C1_Conf_delGPP2_for | acg ctc gga aaa act aca tag ctg |
| C4_Conf_delGPP2_rev | cca cga gct cag aaa tta tgt ttg |
| N1_GPD1_for | cat tat aat att agc tgt aca cct ctt ccg cat ttt ttg aTT TTT TAT TCT TTT TTT TGA |
| N2_GPD1 _rev | att tat tgg aga aag ata aca tat CGC AAA AAA GGG AAT AAG GGC GAC |
| N3_GPD1_for | GTC GCC CTT ATT CCC TTT TTT GCG ata tgt tat ctt tct cca ata aat |
| N4_GPD1_rev | c ttt ata tta tca ata ttt gtg ttt ATG TCT GCT GCT GCT GAT AG |
| N5_GPD1_rev | ggg caa gct ctt tgg aac ttg tcc att tct taa aga cat act tta tat tat caa t |
| C1_Conf_delGPD1_for | cgc ctt gct tct ctc ccc ttc ctt |
| C4_Conf_delGPD1_rev | cac cta ttt cga aaa tga aaa ctc |
| N1_GPD2_for | ttc tac aac tac tac tag taa cat tac tac agt tat tTT TTT TAT TCT TTT TTT TGA |
| N2_GPD2 _rev | TGC AGA GAA GTT CAC GAG TGG CTA CGC AAA AAA GGG AAT AAG GGC GAC |
| N3_GPD2_for | GTC GCC CTT ATT CCC TTT TTT GCG TAG CCA CTC GTG AAC TTC TCT GCA |
| N4_GPD2_rev | t gat aag gaa ggg gag cga aaa ATG CTT GCT GTC AGA AGA TT |
| N5_GPD2_rev | ggc gat tat tta att gtg tta gtg tac agg gtg tcg tct tta tat tat caa t |
| C1_Conf_delGPD2_for | cgg acc tat tgc cat tgt tat tcc |
| C4_Conf_delGPD2_rev | cag att aca cta tct gcc gct acg |
